# Intestinal Microbiota and Serum Metabolic Profile Responded to Two Nutritional Different Diets in Mice

**DOI:** 10.3389/fnut.2021.813757

**Published:** 2022-01-05

**Authors:** Zhifeng Wu, Wei Cheng, Zhenyu Wang, Shuaifei Feng, Huicong Zou, Xiang Tan, Yapeng Yang, Yuqing Wang, Hang Zhang, Miaomiao Dong, Yingping Xiao, Shiyu Tao, Hong Wei

**Affiliations:** ^1^College of Animal Sciences and Technology, Huazhong Agricultural University, Wuhan, China; ^2^State Key Laboratory of Animal Nutrition, College of Animal Science and Technology, China Agricultural University, Beijing, China; ^3^State Key Laboratory for Managing Biotic and Chemical Threats to the Quality and Safety of Agro-products, Institute of Agro-product Safety and Nutrition, Zhejiang Academy of Agricultural Sciences, Hangzhou, China

**Keywords:** germ-free diet, nutrient, microbiota, metabolism, gut development

## Abstract

There is an interaction and bidirectional selection between dietary intake and gut microbiota due to the different efficiency of nutrients in the gut. The nutritional composition of germ-free (GF) diets differs significantly from specific pathogen-free (SPF) diets. There is, however, no data revealing how SPF animals from the same microbial background respond to them and if they affect the host. We examined the growth of SPF mice on the GF diet and found that it reduced body weight, intestinal length and intestinal morphology. Interestingly, the GF diet increased the level of pro-inflammatory bacteria in the gut of SPF mice, including Proteobacteria, *Burkholderiaceae, Alloprevotella* and *Parasutterella*. Furthermore, GF diets caused significant increases in malondialdehyde (MDA), IL-1β, IL-6, and D-lactate levels in the serum of SPF mice and significantly altered their serum metabolic profile, especially amino acid metabolism. In conclusion, GF diets are not suitable for the growth and development of SPF mice. These findings, based on the role of gut microbiota in diet selection, provide new insights into the scientific and rational use of experimental animal diets.

## Introduction

Diets are composed of different types and levels of nutrients, which act differently and can cause considerable changes in the organism. In detail, nutrient deficiencies or excesses affect hormones, metabolic pathways, gene expression, and the composition and function of gut microbes, altering the physiology of the host and having a major impact on growth, reproduction, and metabolism (1). For example, high dietary fat can disrupt the intestinal barrier and lead to inflammation, which is associated with the development of many diseases such as metabolic disorders and cancer (2–4). In animals, specific components of the diet such as dietary fiber enhance sow performance and improve piglet growth through the gut microbiota (5, 6). Therefore, the selection of diets with different nutrient contents and types in animal experiments may lead to bias in the baseline data of the experimental animals themselves due to different nutrient supply and animal needs, thus affecting the scientificity and rationality of the experimental data (7, 8).

The gut microbiota is the largest symbiont community in the body and is considered as an additional organ which closely related to immunity, nutrient absorption and metabolism and other physiological functions (9, 10). The gut microbiota uses nutrients from the diet as substrates for metabolism, and different microbes utilize the same diet differently. In turn, diet regulates the structure of the gut microbiota, so that different diets lead to microbial differences in the same host background (11). Such diet-microbiota interactions have beneficial or detrimental effects on the host by directly altering the microbial structure or by indirectly altering microbial metabolites (12). Microorganisms in the gut produce a large number of small molecules through primary and secondary metabolic pathways, many of which are dependent on the host's diet. The most widely studied of these are short-chain fatty acids (13). Short-chain fatty acids are produced by fermentation of indigestible foods by gut microorganisms and play an important role in providing energy, maintaining intestinal health, and fighting inflammation (14–16). Several studies have shown the correlation between gut microbiota and host physiological functions. Anaerobic bacteria in the intestine form a biological barrier to maintain the normal function of the intestinal mucosa. The intestinal barrier serves as a physical and immune defense against toxins, food antigens, and harmful microbes in the intestinal lumen (17, 18). Intestinal microbial dysbiosis leads to the impaired intestinal barrier. Furthermore, dysbiosis of the gut microbiota causes alterations of metabolites, which translocate from the gut across a disrupted intestinal barrier to affect various metabolic organs, leading to metabolic inflammation and oxidative stress and ultimately to disease (19, 20).

In addition to the regulation of host physiological functions, the gut microbiota ferments certain components of the diet to produce nutrients the host needs such as vitamin K, biotin, pantothenic acid, and pyridoxine (21). As one of the most important experimental models for studying gut microbiota, germ-free (GF) animals do not have the ability to synthesize these nutrients (22, 23). Unlike specific pathogen-free (SPF) animals, which do not have specific pathogens but a complete gut microbiota, GF animals do not contain any microorganisms in their bodies. Therefore, in contrast to SPF animals, the ability of GF animals to utilize nutrients is compromised (24). The GF and SPF diets are recognized as diets that are suitable for two different animals and meet their respective nutritional requirements. Based on the different physiological characteristics and nutritional needs of GF and SPF animals, the composition of their diets is different. For example, The GF diet should be as low in fiber as possible and nutrients such as vitamins, minerals, and amino acids should be supplemented additionally compared to the SPF diet (24, 25). Our laboratory has been studying the diets of GF animals and has formulated diets that meet the growth and reproductive needs of GF mice (26). The GF diet has a higher nutritional content than the SPF diet, both as previously reported and in the formulas we have created.

Based on the comparison and speculation of the nutrient composition of the two different diets, we propose the scientific hypothesis that the gut microbiota in the same host context can lead to microbial selectivity for diet due to differences in nutrient preferences, thus causing differences in a range of physiological functions in the host. Therefore, in this study, we used SPF mice of the same host microbial background (same strain) as animal models to investigate the response of the gut microbiota to the different diets and the differences in metabolism, gut development, inflammatory and oxidative status and growth due to the different response profiles.

## Methods and Materials

### Mice

Sixteen 3-week-old male SPF Kunming (KM) mice were purchased from the Experimental Animal Center of Huazhong Agricultural University (Wuhan, China). Mice were housed in a pathogen-free colony (temperature, 25 ± 2°C; relative humidity, 45–60%; lighting cycle, 12 h/day; light hours 06:30–18:30) with free access to food and water. All animal experiments and sample collection procedures were approved by the Institutional Animal Care and Use Committee of Huazhong Agricultural University, Hubei, China. All experimental methods in this study were carried out following the Guide for the Care and Use of Laboratory Animals at Huazhong Agricultural University. The animal experiment ethics number for this study is HZAUMO-2021-0187.

### Experiment Design and Sample Collections

Mice from the same genetic and microbiological background were divided randomly into 2 groups (*n* = 8/group): (i) mice were fed SPF diet; (ii) mice were fed GF diet. The SPF diet (lot number: 21053113) was purchased in Keao Xieli Feed Co., Ltd. (Beijing, China). The GF diet is manufactured according to our specially designed formula and sterilized by 50 kGy of Co60-γ irradiation to completely kill the microorganisms (27, 28). The GF and SPF diets had the same ingredients but different ratios, and both were tested for the nutrient content according to standards before starting to feed the mice. Table 1 lists the composition and content of both diets. After 7 weeks, mice were sacrificed to collect the duodenum, jejunum, and ileum after the mice were euthanized with CO_2_ inhalation followed by cervical dislocation to ensure death. Full blood samples of mice were collected by extirpating eyeballs. The serum was prepared as follows: the whole blood was left at room temperature for 60 min and then centrifuged (3,500 rpm, 15 min) to remove any remaining insoluble material. The serum is then stored at −80°C. The lengths of the small intestine and colon were measured and the duodenum, jejunum and ileum segments obtained were fixed in 4% paraformaldehyde for hematoxylin-eosin staining. Before sacrifice, fresh feces from each mouse were collected for the microbial sequencing. Measurement of mice weight at the beginning of the experiment (3 weeks old) and before sampling (10 weeks old).

**Table 1:** Ingredient composition of the GF diet and SPF diet.

	**Nutrient levels**	**GF diet**	**SPF diet**		**Nutrient levels**	**GF diet**	**SPF diet**
Nutrient level	Crude protein, g/kg	227.10	231.00	Amino acid	Lysine, g/kg	9.50	13.90
	Crude fat, g/kg	66.50	48.00		Tryptophan, g/kg	1.20	2.50
	Crude fiber, g/kg	22.00	41.00		Arginine, g/kg	12.60	12.00
	Crude ash, g/kg	55.50	70.00		Leucine, g/kg	14.25	17.60
	Moisture, g/kg	69.00	79.00		Isoleucine, g/kg	6.00	11.00
	Energy, Kcal/kg	3820.00	3440.00		Threonine, g/kg	8.00	9.00
Vitamins	Vitamin A, IU/kg	9555.00	20000.00		Valine, g/kg	9.40	11.90
	Vitamin D, IU/kg	1445.00	1667.00		Histidine, g/kg	4.35	5.60
	Vitamin E, mg/kg	82.70	182.00	Minerals	Calcium, g/kg	10.70	12.10
	Vitamin K, mg/kg	2.03	8.00		Total phosphorus, g/kg	11.40	7.90
	Vitamin B1, mg/kg	8.05	20.23		Natrium, g/kg	0.09	2.83
	Vitamin B2, mg/kg	12.60	20.00		Magnesium, g/kg	2.30	2.80
	Vitamin B6, mg/kg	10.80	15.00		Potassium, g/kg	0.70	8.20
	Vitamin B12, mg/kg	0.03	0.03		Copper, mg/kg	18.00	12.41
	Niacin, mg/kg	58.80	70.00		Iron, mg/kg	156.0	158.6
	Pantothenic Acid, mg/kg	25.30	25.00		Manganese, mg/kg	426.50	88.10
	Biotin, mg/kg	0.16	0.30		Zinc, mg/kg	138.00	50.70
	Folic Acid, mg/kg	4.62	10.0		Iodine, mg/kg	0.03	0.90

### Hematological Parameters Testing

Whole blood was collected in 5 mL EDTA anticoagulation tubes. Hematology analyzer VETSCAN HM5 (Abaxis, Inc., Union City, CA, USA) was used to test hematological parameters. The following hematological parameters were measured: white blood cell (WBC), lymphocyte count (LYM), monocyte count (MON), neutrophil count (NEU), red blood cell (RBC), hemoglobin (HGB), hematocrit (HCT), mean cell volume (MCV), mean cell hemoglobin (MCH), mean cell hemoglobin concentration (MCHC), red cell distribution width (RDW), platelet count (PLT), mean platelet volume (MPV), thrombocytosis (PCT) and platelet distribution width (PDW). The lymphocyte ratio (LYM%), monocyte ratio (MON%) and neutrophil ratio (NEU%) were calculated from the above assay values.

### Hematoxylin-Eosin Staining and Analysis

After fixation with 4% paraformaldehyde for 24 h, the duodenum, jejunum, and ileum samples were embedded in paraffin, sectioned and stained with hematoxylin for histological analysis. Determination of villus height and crypt depth were performed using CaseViewer software (version 220 2.2) at 200× magnification. Three tissue sections from each mouse were coded and examined by 2 professionals to prevent observer bias.

### Growth, Intestinal Permeability, Immune and Oxidative Stress Markers Testing

The ELISA kits (Shanghai Enzyme-linked Biotechnology, Shanghai, China) were used to detect various types of indicators in serum including growth-related hormones, inflammatory factors, oxidative stress indicators, and intestinal barrier indicators, according to the manufacturer's instructions. Growth-related hormones included Growth hormone (GH) and Insulin-like growth factor 1 (IGF-1). Inflammatory cytokines included tumor necrosis factors-α (TNF-α), interleukin-1β (IL-1β), interleukin-6 (IL-6), and interleukin-8 (IL-8), oxidative stress indicators included superoxide dismutase (SOD) and malondialdehyde (MDA), diamine oxidase (DAO) and D-lactate (D-LA) were indicators of intestinal permeability. Besides, two immunoglobulins, Immunoglobulin A (IgA) and Immunoglobulin G (IgG) were also measured in serum.

### Bacterial DNA Extraction, 16S rRNA Gene Amplification and Sequencing

Fecal samples were collected and immediately frozen at −80°C. Total DNA was extracted from each fecal specimen by using the QIAamp R Fast DNA Stool Mini Kit (Qiagen Ltd., Germany) following the manufacturer's instructions. The V3–V4 region of the 16S rRNA gene was amplified with primers: 338F (5′-ACTCCTACGGGAGGCAGCA-3′) and 806R (5′-GGACTACHVGGGTWTCTAAT-3′). The amplified products were detected using agarose gel electrophoresis (2% agarose), recovered by AxyPrep DNA Gel Recovery Kit (Axygen Biosciences, Union City, CA, United States), and then quantified by Qubit 2.0 Fluorometer (Thermo Fisher Scientific, Waltham, MA, United States) to pool into equimolar amounts. Paired-end library was constructed using NEXTFLEX Rapid DNA-Seq (Bioo Scientific, Austin, TX, USA) and MiSeq Reagent Kit v3 (Illumina, San Diego, CA, USA) were used for sequencing. Amplicon libraries were sequenced on the Illumina MiSeq 2500 platform (Illumina, San Diego, CA, USA) for paired-end reads of 250 bp. The raw reads were deposited into the NCBI Sequence Read Archive database (accession number: PRJNA768608): https://dataview.ncbi.nlm.nih.gov/object/PRJNA768608. The specific information of the raw sequencing data were listed in Supplementary Table S1.

### Detection of Serum Metabolomics

The untargeted metabolomics profiling was performed on XploreMET platform (Metabo-Profile, Shanghai, China). Briefly, samples were thawed and centrifuged to separate the fragments. Mix 50 μl of sample and 10 μl of internal standard and add 175 μL of pre-cooled methanol/chloroform. After centrifugation 200 μl of supernatant was transferred to an autosampler vial (Agilent Technologies, Foster City, CA, USA). The sample was evaporated using a CentriVap vacuum concentrator (Labconco, Kansas City, MO, USA) to remove the chloroform and the sample was further freeze-dried. Dried samples were derivatized with 50 μL of methoxylamine (20 mg/mL pyridine) for 2 h at 30°C, followed by the addition of 50 μL of MSTFA (N-methyl-N-(trimethylsilyl)trifluoroacetamide) with 1% trimethylchlorosilane (TMCS) containing fatty acid methyl ester (FAMEs) as a retention index at 37°C. The samples were left for a further 1 h at 5°C using the sample preparation head. In the meantime, the derivatized samples were injected with a sample injection tip. Each sample was introduced onto a time-of-flight mass spectrometry (GC-TOF/MS) system (Pegasus) with an Agilent 7890B gas chromatograph for GC-TOFMS analysis.

### Statistical Analysis

16S raw sequencing reads were demultiplexed according to sample-specific barcode (6–8 nucleotides) and imported into the QIIME2 platform (version 2020.2) (29). Quality control and denoising were performed simultaneously using DADA2 with default parameters to generate ASVs (30). All ASVs were classified against the silva 132 database by naïve Bayes classifier constructed by scikit-learn software (31). α-and β-diversity were calculated using the vegan package (version 2.5-6) inside R. PCoA was performed using weighted Bray-Curtis distance metrics. PERMANOVA was used to evaluate factors shaping microbiota by using the adonis function of the “vegan” package (999 permutations). Differential taxa were identified by LefSe (32) and function prediction was performed with PICRUSt2 (version 2.4.1) (33).

The metabolomics raw data were processed using ChromaTOF (V4.71, Leco Corp., St. Joseph, MO, USA) for automatic baseline denoising and smoothing, peak picking, deconvolution, and peak alignment. Compound identification was performed by comparing MS similarity and FAMES retention index distances with reference standards in JiaLib. Statistical analysis includes multivariate statistical analysis such as principal component analysis (PCA), partial least squares discriminant analysis (PLS-DA), etc., and univariate statistical analysis including Student *t*-test, Mann-Whitney-Wilcoxon (*U*-test), etc. All *P*-values were adjusted for false discovery rate (FDR). Statistical algorithms were performed using the widely used statistical analysis package in R Studio (http://cran.r-project.org/).

The differential bacteria and metabolites screened by lefSe were combined according to the group and imported into R. Rcorr function was used to calculate the Pearson correlation coefficient and *P*-value. The differential bacteria were classified by group and metabolites were classified by class, and the classified data were imported into R for heat mapping using the “Pheatmap” package (1.0.8).

All data were presented as means ± SEM. Data were tested for normal distribution and statistical significance was assessed by the independent sample *t*-test using SPSS (SPSS version 20.0 for Windows; SPSS Inc., Chicago, IL, USA) software. Data were considered statistically significant when *P* < 0.05.

## Results

### GF Diet Intake Reduces Body Weight and Shortens the Length of the Small Intestine and Colon in SPF Mice

**Figure 1:**
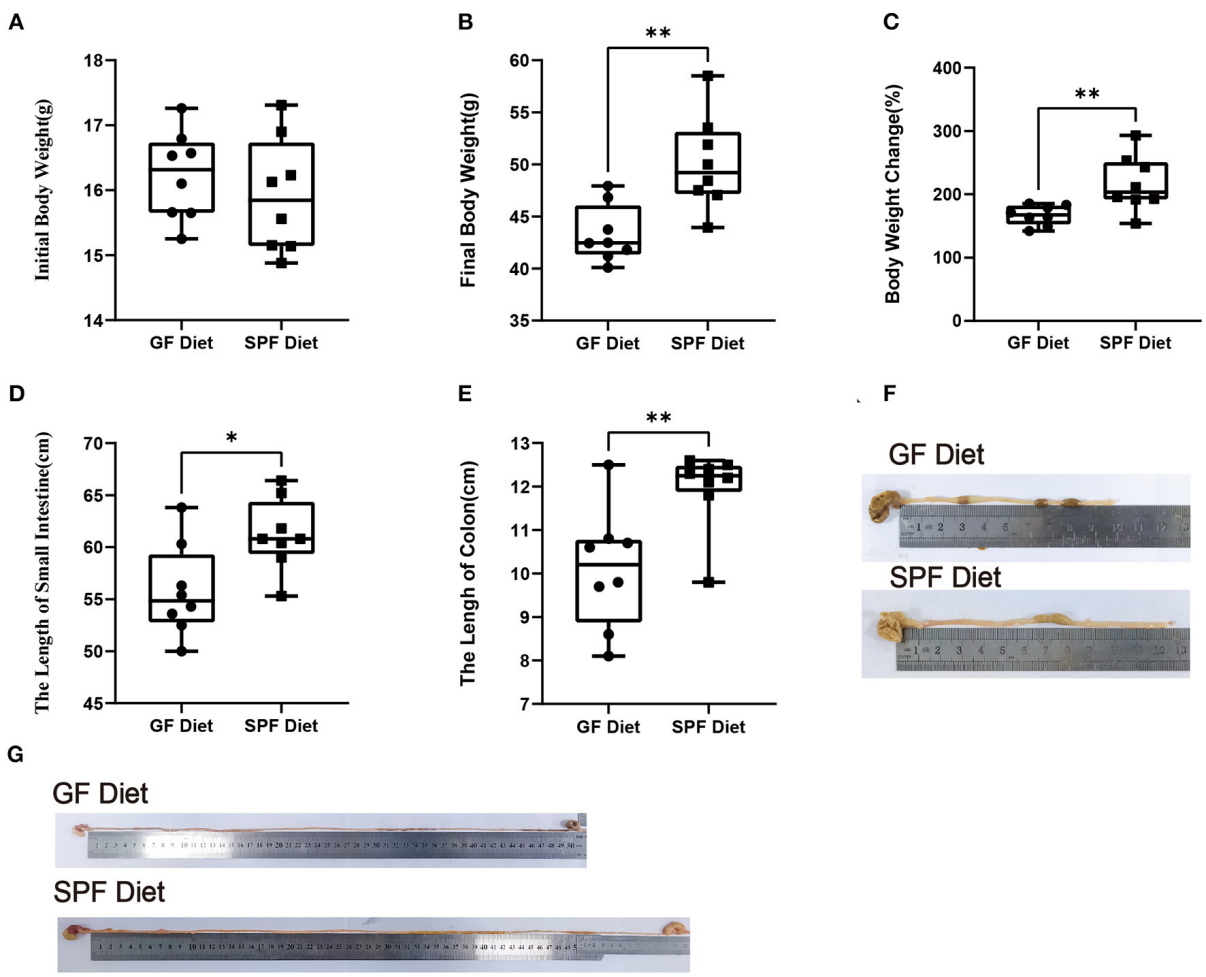
Effect of GF diet on body weight and small intestine and colon length of SPF mice. **(A)** Initial body weight, **(B)** Final body weight, **(C)** Body weight change (%), **(D)** Small intestinal length, **(E)** Colonic length, **(F)** Colonic illustrate, and **(G)** Small intestinal illustrate. The unpaired *t*-test was used to determine whether differences existed between the two groups, *P < 0.05, **P < 0.01. All data are shown as mean ± SEM (*n* = 8).

In the present experiment, the initial body weights of the SPF diet and GF diet group were almost identical (*P* > 0.05; Figure 1A). Compared with SPF diet mice, GF diet mice had significantly lower final body weight and rate of weight change (*P* < 0.01; Figures 1B,C) and significantly shorter lengths of the small intestine (*P* < 0.05; Figures 1D,G) and colon (*P* < 0.01 Figures 1E,F).

### GF Diet Intake Causes Deterioration of Intestinal Morphology in SPF Mice

**Figure 2:**
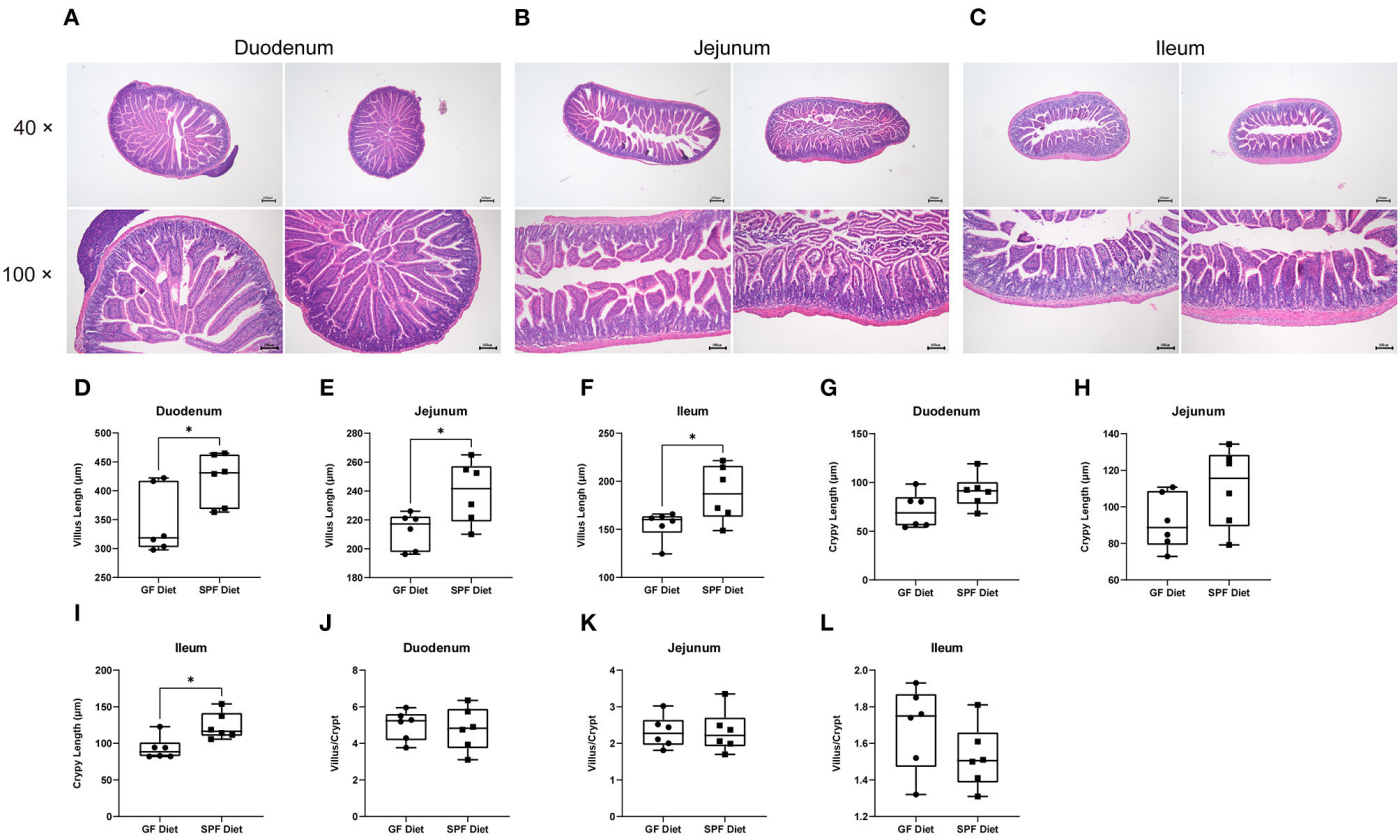
Effect of GF diet on intestinal morphology of SPF mice. Hematoxylin and eosin staining of **(A)** Duodenum, **(B)** Jejunum, and **(C)** Ileum. **(D)** Villus length of duodenum. **(E)** Villus length of jejunum, **(F)** Villus length of ileum, **(G)** Crypt length of duodenum. **(H)** Crypt length of jejunum, **(I)** Crypt length of ileum, **(J)** Villus/Crypt analysis of duodenum. **(K)** Villus/Crypt analysis of jejunum, **(L)** Villus/Crypt analysis of ileum. The unpaired *t*-test was used to determine whether differences existed between the two groups, *P < 0.05. Data are shown as mean ± SEM (*n* = 6).

To investigate the effect of the GF diet on the intestinal morphology of mice, the duodenum, jejunum and ileum were stained with HE (Figures 2A–C). Compared with SPF Diet, the villi length of the duodenum, jejunum and ileum of GF diet mice was significantly lower (*P* < 0.05; Figures 2D–F). The crypt depth of ileum was reduced (*P* < 0.05; Figure 2I) and there was no change in duodenal and jejunal crypt depth (*P* > 0.05; Figures 2G,H). No difference in villi length/crypt depth ratio in duodenum, jejunum and ileum (*P* > 0.05; Figures 2J–L).

### GF Diet Intake Alters Hematological Parameters in SPF Mice

Hematological parameters were examined using anticoagulated blood from mice on the GF diet and SPF diet groups. The results revealed that LYM% and RDWs were significantly lower and MON, MON% and NEU% were significantly higher in the GF diet group compared to the mice in the SPF diet group (*P* < 0.05; Table 2), with no differences in WBC, LYM, NEU, RBC, HGB, HCT, MCV, MCH, MCHC, RDWc, PLT, MPV, PCT, PDWc, and PDWs (*P* > 0.05; Table 2).

**Table 2:** Effect of GF diet and SPF diet on hematological parameters in mice.

**Index/unit**	**GF diet**	**SPF diet**	***P*-value**
WBC/10^9^/L	3.40 ± 1.77	3.45 ± 1.26	0.948
LYM/10^9^/L	2.15 ± 1.67	2.52 ± 0.81	0.589
LYM%/%	57.73 ± 18.46	73.50 ± 4.70	0.030
MON/10^9^/L	0.23 ± 0.11	0.13 ± 0.02	0.328
MON%/%	8.23 ± 4.44	4.21 ± 1.40	0.034
NEU/10^9^/L	1.02 ± 0.36	0.80 ± 0.49	0.029
NEU%/%	34.06 ± 14.4	22.26 ± 5.45	0.048
RBC/10^12^/L	9.10 ± 2.58	10.74 ± 1.48	0.139
HGB/g/dl	11.20 ± 4.08	13.59 ± 1.55	0.144
HCT/%	42.40 ± 13.06	51.58 ± 5.78	0.090
MCV/fl	46.63 ± 3.46	48.13 ± 2.59	0.343
MCH/pg	12.00 ± 1.83	12.73 ± 1.10	0.353
MCHC/g/dl	25.81 ± 3.83	26.38 ± 1.62	0.708
RDWc/%	19.00 ± 0.76	19.48 ± 1.56	0.452
RDWs/fl	33.21 ± 1.93	35.36 ± 1.29	0.020
PLT/10^9^/L	509.88 ± 145.31	569.88 ± 175.30	0.468
MPV/fl	7.45 ± 1.23	6.98 ± 0.33	0.309
PCT/%	0.39 ± 0.17	0.40 ± 0.13	0.884
PDWc/%	33.14 ± 3.45	31.39 ± 2.58	0.270
PDWs/fl	11.83 ± 4.05	10.08 ± 2.05	0.294

*WBC, White blood cell; LYM, Lymphocyte count; LYM%, Lymphocyte ratio; MON, Monocyte count; MON%, Monocyte ratio; NEU, Neutrophil count; NEU%, Neutrophil ratio; RBC, Red blood cell; HGB, Hemoglobin; HCT, Hematocrit; MCV, Mean cell volume; MCH, Mean cell hemoglobin; MCHC, Mean cell hemoglobin concentration; RDWc, Red cell distribution width (CV), RDWs, Red cell distribution width (SD); PLT, Platelet count; MPV, Mean platelet volume; PCT, Phrombocytosis; PDWc, Platelet distribution width (CV); PDWs, Platelet distribution width (SD)*.

### GF Diet Intake Alters Intestinal Permeability and Induces Inflammatory Responses

**Figure 3:**
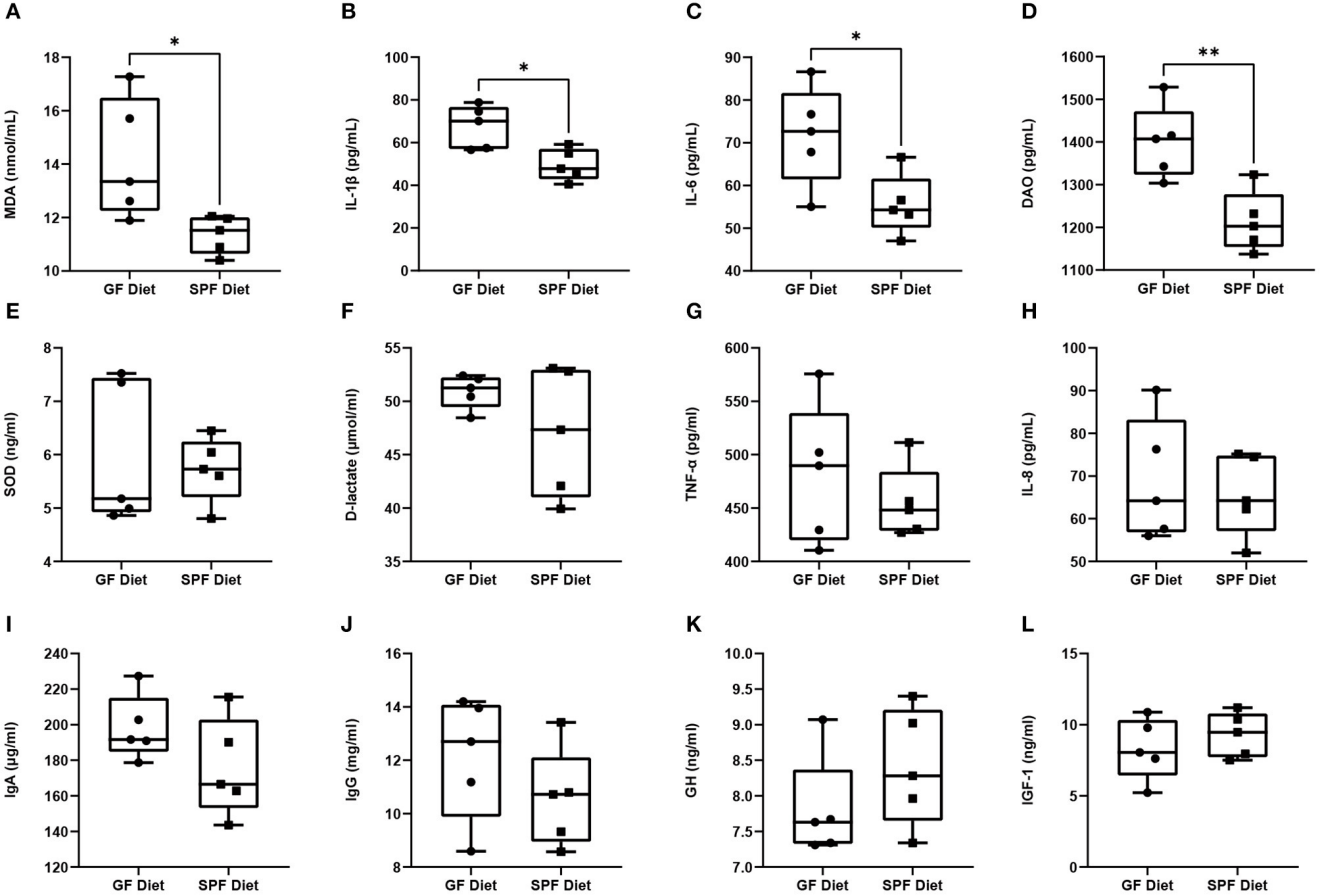
Effect of GF diet on intestinal permeability, inflammatory response, oxidative stress, hormones and immune factors. **(A)** Malondialdehyde (MDA), **(B)** Interleukin-1β (IL-1β), **(C)** Interleukin-6 (IL-6), **(D)** Diamine oxidase (DAO), **(E)** Superoxide dismutase (SOD), **(F)** D-lactate (D-LA), **(G)** Tumor necrosis factors-α (TNF-α), **(H)** Interleukin-8 (IL-8), **(I)** Immunoglobulin A (IgA), **(J)** Immunoglobulin G (IgG), **(K)** Growth hormone (GH), and **(L)** Insulin-like growth factor 1 (IGF-1) contents in serum of SPF mice. Values are presented as mean ± SEM (*n* = 5). Statistical significance was calculated using unpaired *t*-test, *P < 0.05; **P < 0.01.

To investigate the effects of the GF diet on growth, immunity, inflammation, oxidative stress, and intestinal permeability in SPF mice, we measured the serum parameters. The levels of MDA, IL-1β, IL-6, and D-lactate were found to be significantly higher in the GF diet mice than in the SPF diet (*P* < 0.05; Figures 3A–D), while the remaining indicators were not statistically different (*P* > 0.05; Figures 3E–L).

### GF Diet Intake Changes the Fecal Microbiota of SPF Mice

**Figure 4:**
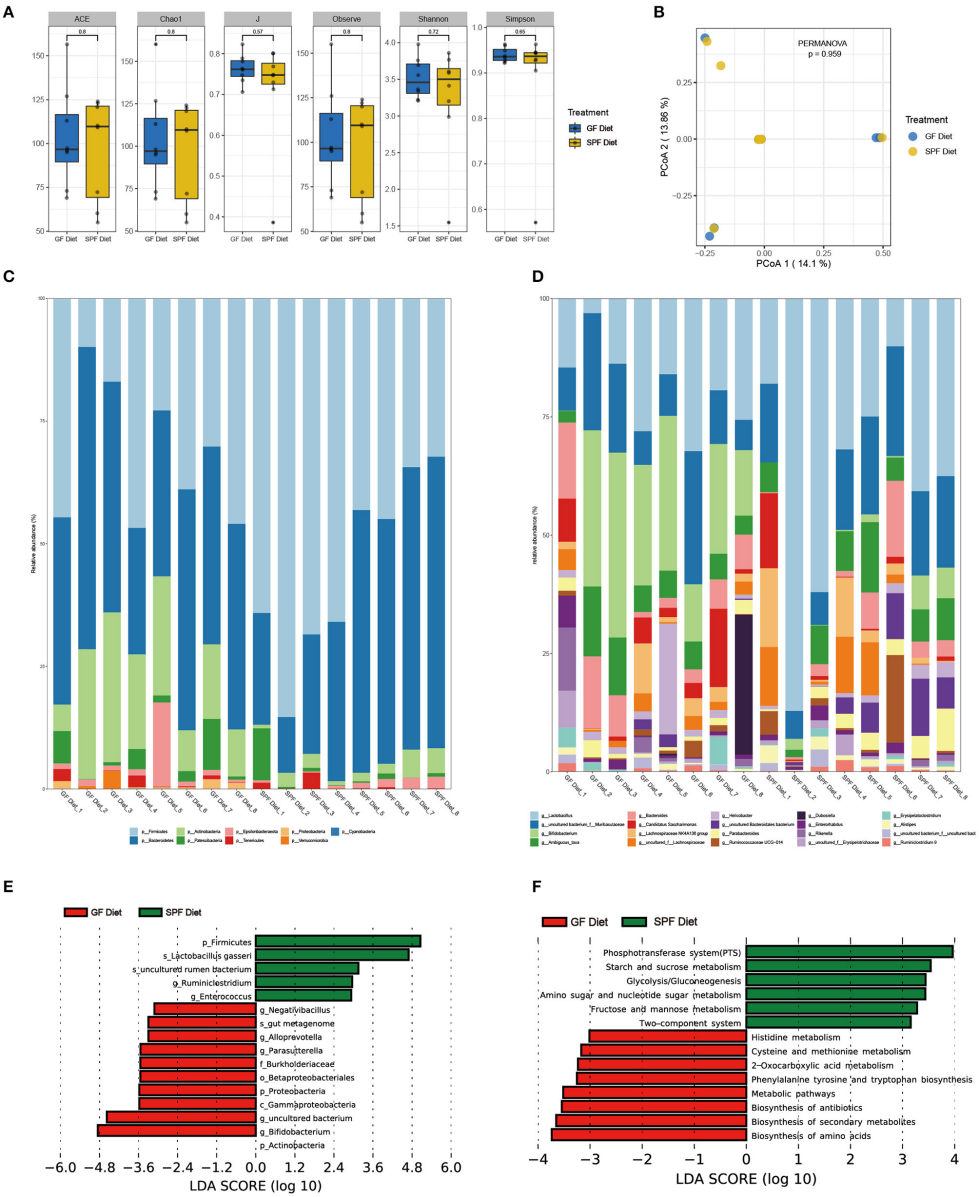
Effect of GF diet on fecal microbial community of SPF mice. **(A)** Fecal microbial α-diversity. **(B)** Principal coordinates analysis (PCoA) based on the total OTUs. The phylum **(C)** and the genus **(D)** in relative abundance of the fecal microbiota. **(E)** Identification of differential bacterial taxa by LefSe tool (LDA score > 2.0). The length of the column is proportional to the taxa abundance. **(F)** Assess the functional content of microbiota.

Feces from two groups of mice were collected to examine the differences in fecal microbiota between the GF diet and SPF diet group. Assessment of microbiota composition and diversity of mouse fecal samples by deep sequencing of the V3–V4 region of the 16S rRNA gene. A total of 844,103 high-quality 16S rRNA gene sequences were obtained from 16 fecal samples. The average number of high-quality sequences generated per sample was 52,756. The microbial α-diversity in the fecal samples of both groups is shown in Figure 4A. The observed fecal microbial species, Chao1, ACE, Shannon, Simpson, and J indices did not change significantly between the SPF diet and GF diet group (*P* > 0.05; Figure 4A). PCoA based on Bray-Curtis distance showed that the structure of the fecal microbial community was similar in both groups of mice (Figure 4B). The relative abundance of fecal microbiota levels indicated that Firmicutes, Bacteroidetes, and Actinobacteria were predominant in the feces of both groups of mice (Figure 4C). At the genus level, as shown in Figure 4D, *Lactobacillus, uncultured bacterium, Bifidobacterium*, and *Bacteroides* were the dominant genera in both groups of mice (Figure 4D). Using LefSe analysis of gut microbial abundance in all samples, bacteria with *P* < 0.05 and LDA > 2.0 were screened, and we identified 16 different levels of differential bacteria (*P* < 0.05; Table 3). At the phylum level, the abundance of Firmicutes was reduced and Actinobacteria and Proteobacteria were increased in the GF diet group. At the genus level, the relative abundances of *Negativibacillus, Alloprevotella, Parasutterella, uncultured bacterium*, and *Bifidobacterium* were significantly elevated and *Ruminiclostridium* and *Enterococcus* were significantly reduced in the feces of the GF diet group compared with those from the SPF diet group (Figure 4E). Besides, at other different levels, the abundance of *Gammaproteobacteria, Betaproteobacteriales, Burkholderiaceae*, and *gut metagenome* increased significantly in the GF diet group, while *uncultured rumen bacterium* and *Lactobacillus gasseri* decreased significantly (Figure 4E).

**Table 3:** All levels of differential bacteria in the feces between the GF diet group and the SPF diet group.

**Microbial taxa**	**LDA**	**Enrichment**	***P*-value**
p_Actinobacteria	4.865	GF diet	0.001
p_Firmicutes	5.055	SPF diet	0.046
p_Proteobacteria	3.575	GF diet	0.013
*c_Gammaproteobacteria*	3.577	GF diet	0.007
*o_Betaproteobacteriales*	3.541	GF diet	0.027
*f_Burkholderiaceae*	3.541	GF diet	0.027
*g_Alloprevotella*	3.295	GF diet	0.027
*g_Bifidobacterium*	4.852	GF diet	0.008
*g_Enterococcus*	2.931	SPF diet	0.011
*g_Negativibacillus*	3.112	GF diet	0.027
*g_Parasutterella*	3.540	GF diet	0.027
*g_Ruminiclostridium*	2.960	SPF diet	0.027
*g_unculted bacterium*	4.571	GF diet	0.036
*s_gut metagenome*	3.295	GF diet	0.027
*s_Lactobacillus gasseri*	4.692	SPF diet	0.027
*s_uncultured rumen bacterium*	3.152	SPF diet	0.008

*p, Phylum; c, Class; o, Order; f, Family; g, Genus; s, Species*.

Next, we used PICRUSt2 to assess the functional content of the microbiota based on the 16S data. A total of 14 Kyoto Encyclopedia of Genes and Genomes (KEGG) Orthology including related to Metabolism and Environmental Information Processing showed differences between GF diet and SPF diet group (*P* < 0.05; Supplementary Table S2). A significantly higher production capacity of biosynthesis of amino acids, biosynthesis of secondary metabolites, biosynthesis of antibiotics, metabolic pathways, phenylalanine, tyrosine and tryptophan biosynthesis, 2-Oxocarboxylic acid metabolism, cysteine and methionine metabolism and histidine metabolism was observed in the GF diet group. However, compared with the SPF diet group, phosphotransferase system (PTS), starch and sucrose metabolism, glycolysis/gluconeogenesis, amino sugar and nucleotide sugar metabolism, fructose and mannose metabolism, the two-component system showed significantly lower in the GF diet group (Figure 4F).

### GF Diet Intake Changes Metabolic Profiles of Serum in SPF Mice

**Figure 5:**
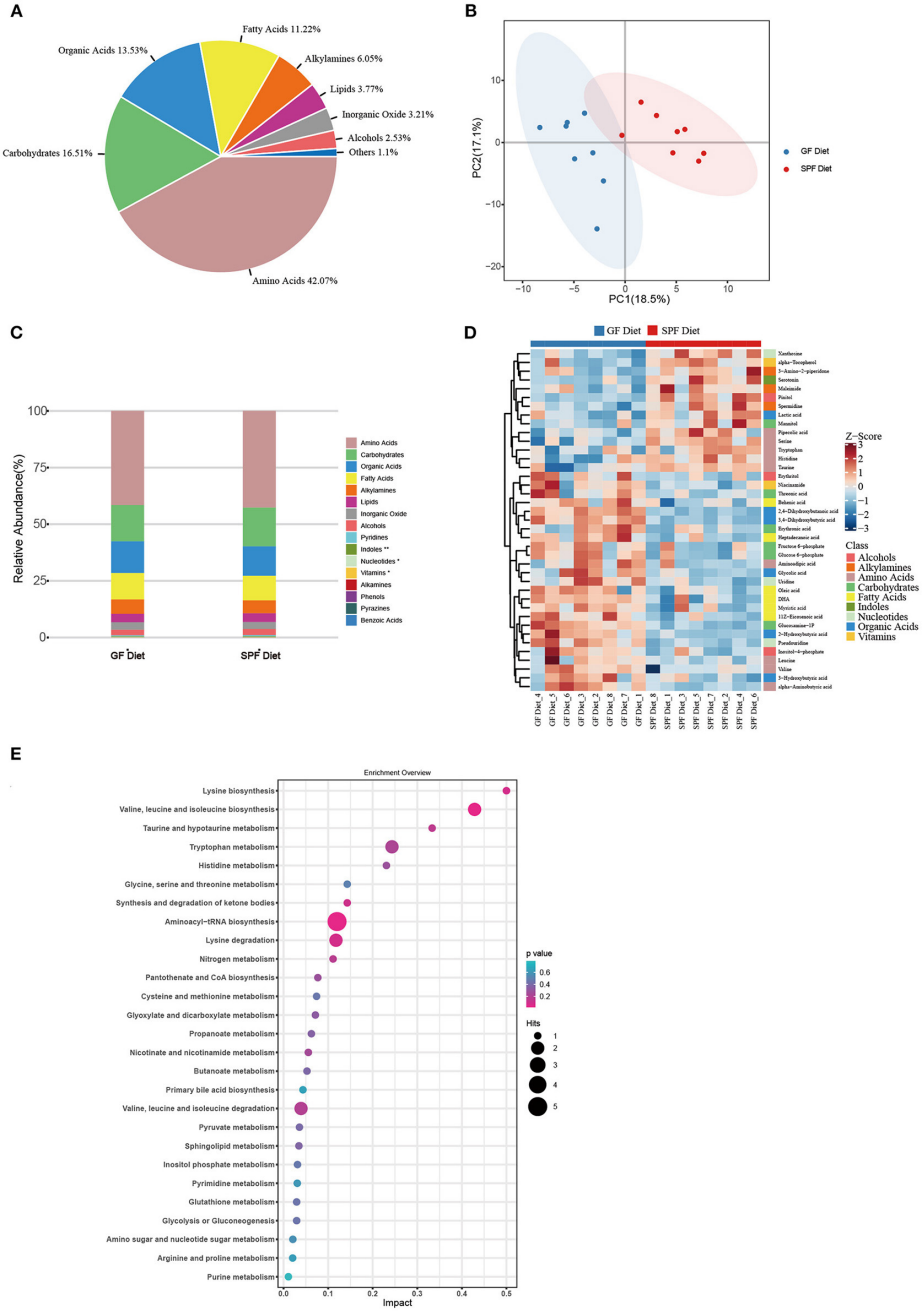
Effect of GF diet on metabolic profiles of serum in SPF mice. **(A)** Distribution of metabolite classes. **(B)** Principal component analysis (PCA) score plot of all samples. **(C)** Distribution of the relative abundance of metabolites of each Class in different groups. **(D)** Heatmap of the potential biomarkers. The relative abundance values of metabolites in different samples are depicted by color intensity. **(E)** Pathway analysis bubble plot. The horizontal coordinate is the extent to which the pathway is affected, and the number of differential metabolites in the pathway is represented by graphs of different sizes. The *P*-values calculated by the enrichment analysis are described in terms of color intensity.

Serum from both groups of mice was collected for non-targeted metabolomic analysis on the GC-TOF/MS platform and a total of 144 metabolites were identified. The classes of metabolites identified and the number of metabolites in each class were shown in Figure 5A. The main metabolites detected were amino acids (42.07%), carbohydrates (16.51%), organic acids (13.53%), and fatty acids (11.22%). PCA was used to observe within- and between-group sample variability and possible outliers. The PCA score plot found a significant difference between the GF diet and SPF diet groups, indicating that the different dietary treatments had a significant effect on serum metabolites (Figure 5B). Analysis of the various metabolites in the two groups of samples revealed significant differences in nucleotide and vitamin-related metabolites (*P* < 0.05; Figure 5C) and extremely significant differences in indole-related metabolites (*P* < 0.01; Figure 5C). Single and multidimensional tests were used to obtain the differential metabolites between the two groups and a total of 39 differential metabolites were identified (*P* < 0.05, Table 4). The major differential metabolites among the different dietary treatments were amino acids and carbohydrates, followed by organic acids and fatty acids, and others (Figure 5D).

**Table 4:** Altered metabolites in the serum between GF diet and SPF diet groups.

**Metabolite**	**Class**	**GF diet**	**SPF diet**	***P*-value**
alpha-Aminobutyric acid	Amino acids	4.40 ± 1.36	2.27 ± 0.43	0.003
Valine	Amino acids	292.13 ± 51.84	202.03 ± 77.99	0.010
Leucine	Amino acids	311.77 ± 53.72	266.14 ± 19.39	0.028
Serine	Amino acids	81.38 ± 24.34	123.96 ± 15.43	0.002
Taurine	Amino acids	208.34 ± 134.03	358.64 ± 28.76	0.005
Aminoadipic acid	Amino acids	1.52 ± 0.50	1.02 ± 0.35	0.050
Histidine	Amino acids	16.75 ± 14.61	33.27 ± 8.73	0.025
Tryptophan	Amino acids	215.32 ± 18.35	235.34 ± 15.87	0.047
Pipecolic acid	Amino acids	7.93 ± 2.17	12.16 ± 3.08	0.011
Threonic acid	Carbohydrates	3.41 ± 0.89	2.58 ± 0.33	0.028
Erythronic acid	Carbohydrates	51.74 ± 13.75	37.79 ± 8.90	0.044
Glucosamine-1P	Carbohydrates	1.75 ± 0.31	0.84 ± 0.19	0.000
Mannitol	Carbohydrates	18.70 ± 2.89	24.21 ± 4.39	0.017
Fructose 6-phosphate	Carbohydrates	1.42 ± 0.34	0.93 ± 0.43	0.033
Glucose 6-phosphate	Carbohydrates	2.71 ± 0.73	1.53 ± 0.72	0.009
Myristic acid	Fatty acids	10.02 ± 1.18	7.55 ± 2.43	0.036
Heptadecanoic acid	Fatty acids	6.91 ± 1.09	5.14 ± 0.83	0.005
Oleic acid	Fatty acids	187.58 ± 31.14	146.53 ± 22.40	0.007
11Z-Eicosenoic acid	Fatty acids	3.88 ± 0.73	2.65 ± 0.51	0.003
DHA	Fatty acids	1.92 ± 0.14	1.30 ± 0.48	0.010
Behenic acid	Fatty acids	0.56 ± 0.08	0.44 ± 0.08	0.019
Lactic acid	Organic acids	365.47 ± 52.72	440.38 ± 49.46	0.016
Glycolic acid	Organic acids	29.80 ± 7.16	20.71 ± 2.71	0.010
2-Hydroxybutyric acid	Organic acids	7.68 ± 2.16	3.16 ± 0.68	0.000
3-Hydroxybutyric acid	Organic acids	477.18 ± 147	306.36 ± 105.92	0.027
3,4-Dihydroxybutyric acid	Organic acids	1.35 ± 0.20	0.69 ± 0.12	0.000
2,4-Dihydroxybutanoic acid	Organic acids	12.09 ± 1.49	5.88 ± 0.80	0.000
Erythritol	Alcohols	3.32 ± 0.49	2.84 ± 0.29	0.047
Pinitol	Alcohols	0.16 ± 0.05	0.55 ± 0.20	0.000
Inositol-4-phosphate	Alcohols	22.02 ± 2.79	18.63 ± 2.30	0.027
Maleimide	Alkylamines	9.19 ± 1.73	11.42 ± 2.05	0.046
3-Amino-2-piperidone	Alkylamines	3.89 ± 1.31	5.83 ± 1.47	0.021
Spermidine	Alkylamines	0.22 ± 0.10	0.46 ± 0.14	0.004
Pseudouridine	Nucleotides	6.46 ± 0.92	4.24 ± 0.44	0.000
Uridine	Nucleotides	0.80 ± 0.28	0.39 ± 0.16	0.007
Xanthosine	Nucleotides	0.07 ± 0.02	0.12 ± 0.03	0.002
Niacinamide	Vitamins	14.94 ± 2.77	23.49 ± 4.68	0.022
alpha-Tocopherol	Vitamins	4.40 ± 1.36	2.27 ± 0.43	0.010
Serotonin	Indoles	292.13 ± 51.84	202.03 ± 77.99	0.001

For amino acids, the GF diet significantly increased the content of valine, leucine, and aminoadipic acid (*P* < 0.05) and extremely significantly increased the content of alpha-Aminobutyric acid (*P* < 0.01), while significantly decreasing the content of histidine, tryptophan and pipecolic acid (*P* < 0.05) and extremely significantly decreasing the content of serine and taurine (*P* < 0.01) compared with the SPF diet group. For carbohydrates, all metabolites were significantly higher (*P* < 0.05) in the GF diet group compared to the SPF diet group, except for mannitol, which was significantly lower in the GF diet group (*P* < 0.05). For organic Acids, similar to carbohydrates, all metabolites were elevated in the GF diet group except for lactic acid (*P* < 0.05). For fatty acids, all metabolites were increased in the GF diet group compared to the SPF diet (*P* < 0.05).

Through pathway analysis, all metabolites were mapped onto 32 KEGG metabolic pathways (Supplementary Table S3). Removing the pathways with an impact value of 0, 27 KEGG metabolic pathways were finally identified including amino acid metabolism (14 metabolites), carbohydrate metabolism (7 metabolites), metabolism of cofactors and vitamins (2 metabolites), metabolism of other amino acids (2 metabolites), lipid metabolism (2 metabolites), and nucleotide metabolism (2 metabolites) (Figure 5E).

### Correlations Between Gut Microbiota and Metabolites, Hematological Parameters, and Intestinal Length

**Figure 6:**
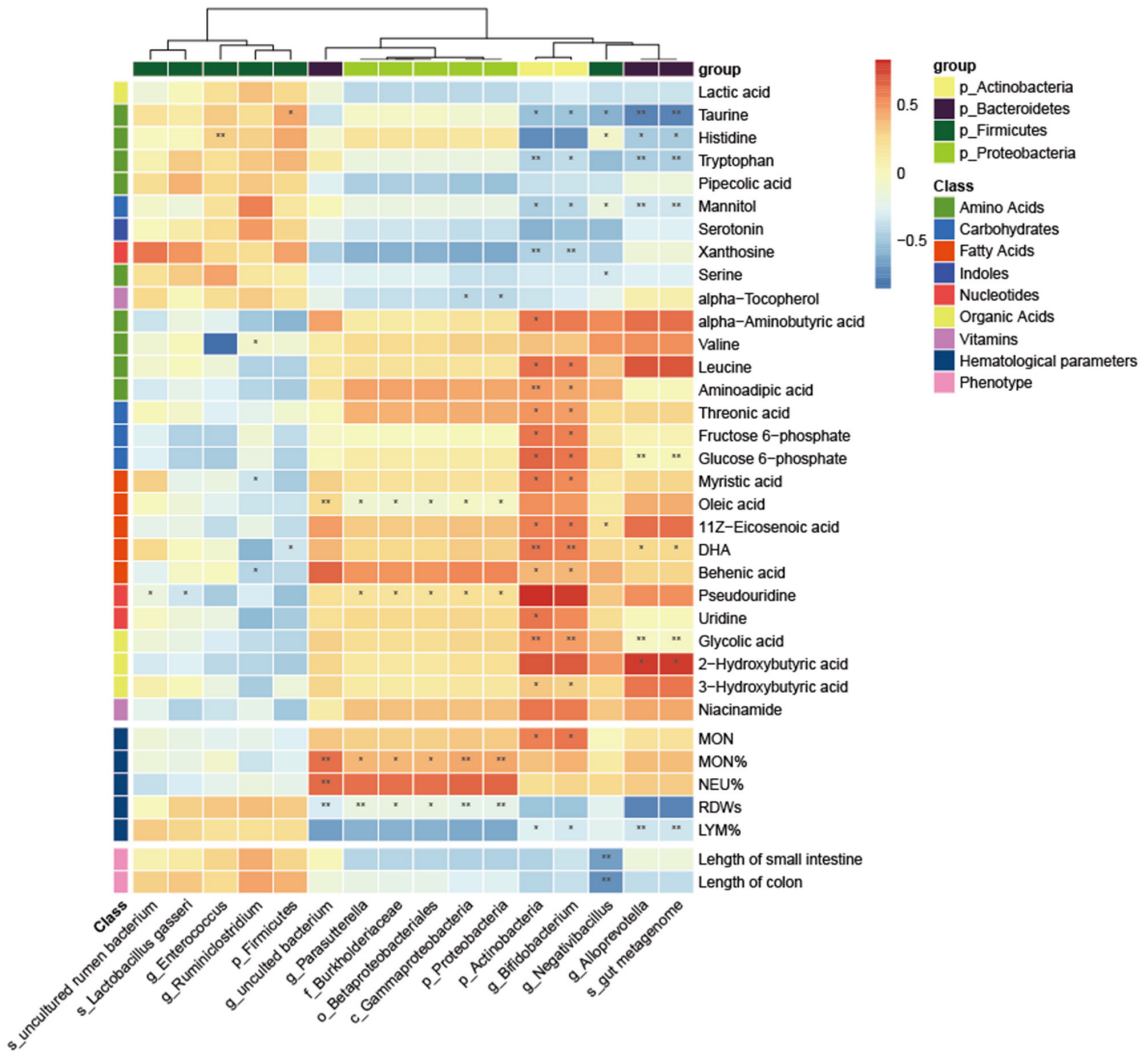
Correlation heatmap between the differential microbiota and specific metabolites, hematological parameters and intestinal length. The Pearson correlation coefficient was used to examine the correlation. *P < 0.05, **P < 0.01.

Next, we screened for differential metabolites by different classes and investigated potential associations between all levels of differential gut microbiota and metabolites, hematological parameters and intestinal length. As shown in the heatmap in Figure 6, Actinobacteria and *Bifidobacterium* ware significantly positively related to leucine, aminoadipic acid, threonic acid, fructose 6-phosphate, glucose 6-phosphate, myristic acid, 11Z-Eicosenoic acid, Behenic acid 3-Hydroxybutyric acid, DHA and glycolic acid while significantly negatively related to taurine, tryptophan, mannitol, and xanthosine. Moreover, another bacterium that significantly affects metabolites is *Alloprevotella*, which was significantly positively to glucose 6-phosphate, DHA, Glycolic acid and 2-Hydroxybutyric acid while significantly negatively related to taurine, histidine, tryptophan, and mannitol. For hematological parameters, Several bacteria from Proteobacteria (*Parasutterella, Burkholderiaceae, Betaproteobacteriales, Gammaproteobacteria*) were significantly positively correlated with MON% and negatively correlated with RDWs. For intestinal length, *Negativibacillus* was significantly negatively related to the length of the small intestine and colon.

## Discussion

Extensive studies of the gut microbiota have shown that diet can regulate the composition and function of the microbial community in the intestine (11, 34–36). Furthermore, diet drives the metabolism of the gut microbiota, making metabolites a link between diet and different physiological states (13, 37, 38). A notable example is that high-fat diet can alter the microbiota structure and metabolic profile of mice, leading to an inflammatory response and impairment of intestinal barrier function (3, 4). In our study, we measured the actual nutrient content in GF and SPF diets. Although we added more nutritional ingredients to the GF diet, we found that the nutrient content of the GF diet was lower than that of the SPF diet. This is because high dose irradiation leads to nutrient loss in the GF diet. Consistent with previous reports, we found that irradiation led to a reduction in vitamins, amino acids, and some minerals in the GF diet (27, 28, 39).

Diets with different nutrient contents lead to differences in the microbiota and metabolic profile of the organism (7, 40). In the present study, we observed differences in gut microbiota and serum metabolism in SPF mice due to GF diet. For gut microbiota, the relative abundance of Firmicutes was reduced and the abundance of Bacteroidetes was increased in the GF diet group. This is possibly due to the low fiber content of the GF diet. Previous studies have shown that the fiber content of the diet correlates with the ratio of Firmicutes/Bacteroidetes, with the lower the fiber content, the higher the Firmicutes/Bacteroidetes ratio (41). Furthermore, we found that the abundance of several inflammation-associated microbiotas was significantly upregulated in the GF diet group, including Proteobacteria, *Burkholderiaceae, Alloprevotella*, and *Parasutterella*. Studies have previously shown that Proteobacteria and *Parasutterella* are associated with dysbiosis of the gut microbiota and chronic inflammation of the gut (42–44). *Burkholderiaceae* is associated with the development of Inflammatory bowel disease (IBD) (45) and *Alloprevotella* associated with metabolic disorders due to unhealthy diet (46). These suggested that the GF diet affects the intestine by increasing harmful bacteria in the gut. By metabolomic analysis, we found that the GF diet resulted in different metabolic patterns in the serum. For specifically affected metabolites, the GF diet increased the content of alpha-aminobutyric acid, valine, leucine, and aminoadipic acid compared with the SPF diet. Increased levels of alpha-aminobutyric acid are thought to be a marker for a range of diseases (47, 48). Valine and leucine are branched-chain amino acids (BCAA) and recent studies have reported a role for BBCA in the development of diseases such as type 2 diabetes (T2D), IBD, and cardiovascular disease (49, 50). Moreover, we observed differences in the KEGG metabolic pathway between the GF diet and SPF diet groups mainly in amino acid metabolism. Besides the low amino acid content observed in GF diet, amino acid imbalance may also have a detrimental effect on SPF mice. Amino acid imbalance causes many diseases of the body (51). Recent studies have shown that imbalance of amino acids, especially the ratio of BCAA to non-BCAA (especially tryptophan and threonine) altered the whole-body metabolism in mice (52). Further analysis revealed that Actinobacteria, *Bifidobacterium, Negativibacillus, Alloprevotella*, and *gut metagenom* were negatively related to the abundance of Taurine, Histidine, and Tryptophan, while Actinobacteria and *Bifidobacterium* were positively correlated with several BCAAs. This suggests that alterations in specific bacteria disrupt the amino acid balance and thus lead to changes in the metabolic profile.

MON are circulating white blood cells that are important in both innate and adaptive immunity and play a major role in immune defense, inflammation and tissue remodeling (53). The numbers of MON increase during acute infection and inflammation (54). We found an increase in the number of MON in the GF diet group. We also measured several serum indicators related to inflammation and found significantly higher levels of IL-1β and IL-6 in the GF diet group. IL-1β and IL-6 are key mediators of the inflammatory response (55). IL-1β affects T cell maturation and the proliferation of B cells. Besides, IL-1β promotes the expression of several inflammatory molecules such as nitric oxide and phospholipase A2 (56, 57). IL-6 is involved in the regulation of the acute phase response to injury and infection. Its dysregulation is associated with the development of various diseases such as IBD, multiple sclerosis and various cancers (58). Our data showed that IL-1β and IL-6 levels were significantly increased in the serum of GF diet mice. The RDW reflects the degree of heterogeneity of erythrocyte volume. The increase in RDW reflects a severe disruption of erythrocyte homeostasis and may be attributable to a variety of underlying metabolic abnormalities such as oxidative stress, inflammation, malnutrition and erythrocyte ruptured (59). We found RDW was significantly increased in the GF diet group of mice. Another indicator of oxidative stress observed to be significantly increased in the GF diet group was MDA. MDA is a metabolic product of free radical-induced peroxidation of unsaturated fatty acids in biological membranes, and its level reflects the degree of lipid peroxidation in the body and indirectly the degree of cellular damage (60, 61). Through correlation analysis, we found that these indicators correlated with the abundance of Proteobacteria. The altered abundance of Proteobacteria is one of the features of gut microbial dysbiosis, and its elevated abundance leads to intestinal epithelial dysfunction and intestinal inflammation (62, 63). These results suggest that the GF diet leads to inflammation and oxidative stress in the organism by increasing the abundance of Proteobacteria in the gut.

In the present study, we found intestinal dysplasia and impairment of intestinal barrier function in mice from the GF diet group. The length of the small intestine and colon in the mice from the GF diet group was extremely significantly shortened. The small intestine is the main digestive organ of the body and is the primary site for nutrient absorption. Villi expand the surface area of the intestine and help the body to absorb nutrients from food. Therefore, a decrease in the length of the villi is associated with decreased nutrient absorption, weight gain and fat accumulation in animals (64). We observed a significant negative correlation between *Negativibacillus* and small intestinal and colonic length. *Negativibacillus* is a pathogenic bacteria associated with gut dysbiosis or pediatric Crohn's disease (65, 66). D-LA, a chemical marker of the intestinal barrier, is present at low levels in healthy individuals, and levels of D-LA increase when the intestinal barrier is disrupted (67). A significant increase in the serum concentrations of D-LA was observed in mice from GF diet, suggesting that GF diet intake induced an increase in intestinal permeability in mice.

Weight loss in SPF mice is attributed to inflammation, intestinal dysplasia and oxidative stress following GF diet intake. The intake of GF diet altered the gut microbiota and serum metabolic profile of mice, and the increase of harmful bacteria in the gut led to damage of the intestinal barrier function (68). Current research suggests that disruption of intestinal barrier function leads to increased intestinal permeability, which facilitates the transport of harmful substances and pathogens to the bloodstream, leading to inflammation and oxidative stress (69, 70). Pathophysiology of various diseases associated with inflammation and oxidative stress. Oxidative stress can promote inflammation, conversely, inflammatory processes also promote oxidative stress and injury. Both of them can cause injury to cells and contribute to a diverse set of pathologies (71, 72). Overall, diet altered the gut microbiota, leading to damage to the intestinal barrier and inflammation, ultimately disrupting the healthy physiological state of the mice.

## Conclusions

In the present study, we found considerable differences in nutrient content between the GF and SPF diets and due to the special nutritional composition, the GF diet altered the structure of the gut microbiota, increased pro-inflammatory bacteria in the gut such as Proteobacteria, *Burkholderiaceae, Alloprevotella*, and *Parasutterella*. In addition, the GF diet altered the serum metabolic profile especially amino acid metabolism in SPF mice. Furthermore, the GF diet was found to cause a significant increase in levels of MDA, IL-1β, IL-6, and D-lactate in the serum and these indicators in the serum indicated that the GF diet destroyed the intestinal barrier, leading to inflammatory responses and oxidative stress, which ultimately led to weight loss in SPF mice. These results are helpful to enhance our understanding of the effects of different nutrient composition diets on host physiological status through the gut microbiota, and also provide new ideas for the scientific selection of diets for experimental animals.

## Data Availability Statement

The datasets presented in this study can be found in online repositories. The names of the repository/repositories and accession number(s) can be found in the article/Supplementary Materials.

## Ethics Statement

The animal study was reviewed and approved by Scientific Ethics Committee of Huazhong Agricultural University.

## Author Contributions

ZWu, WC, and ST designed the experiments. ZWu, XT, YW, and MD carried out the experiments and collected the samples. YY and HZ performed the analysis of samples. ZWa, SF, and HZ analyzed the 16S rRNA and metabolomics data. ZWu drafted the manuscript. ST, YX, and HW revised the manuscript. All authors contributed to the article and approved the submitted version.

## Funding

This work was supported by the National Nature Science Foundation of China (31902189) and the Fundamental Research Funds for the Central Universities (2662020DKQD004, 2662019PY012).

## Conflict of Interest

The authors declare that the research was conducted in the absence of any commercial or financial relationships that could be construed as a potential conflict of interest.

## Publisher's Note

All claims expressed in this article are solely those of the authors and do not necessarily represent those of their affiliated organizations, or those of the publisher, the editors and the reviewers. Any product that may be evaluated in this article, or claim that may be made by its manufacturer, is not guaranteed or endorsed by the publisher.
